# Ethyl Acetate Extract of *Cichorium glandulosum* Activates the P21/Nrf2/HO-1 Pathway to Alleviate Oxidative Stress in a Mouse Model of Alcoholic Liver Disease

**DOI:** 10.3390/metabo15010041

**Published:** 2025-01-10

**Authors:** Shuwen Qi, Chunzi Zhang, Junlin Yan, Xiaoyan Ma, Yewei Zhong, Wenhui Hou, Juan Zhang, Tuxia Pang, Xiaoli Ma

**Affiliations:** 1 College of Pharmacy, Xinjiang Medical University, Urumqi 830011, China; 2 Key Laboratory of Active Components of Xinjiang Natural Medicine and Drug Release Technology, Urumqi 830000, China

**Keywords:** *Cichorium glandulosum*, alcoholic liver disease, oxidative stress, transcriptomics, network pharmacology

## Abstract

Background: Alcoholic liver disease (ALD) is a significant global health concern, primarily resulting from chronic alcohol consumption, with oxidative stress as a key driver. The ethyl acetate extract of *Cichorium glandulosum* (CGE) exhibits antioxidant and hepatoprotective properties, but its detailed mechanism of action against ALD remains unclear. This study investigates the effects and mechanisms of CGE in alleviating alcohol-induced oxidative stress and liver injury. Methods: Ultra-Performance Liquid Chromatography coupled with Quadrupole-Orbitrap Mass Spectrometry (UPLC-Q-Orbitrap-MS) was used to identify CGE components. A C57BL/6J mouse model of ALD was established via daily oral ethanol (56%) for six weeks, with CGE treatment at low (100 mg/kg) and high doses (200 mg/kg). Silibinin (100 mg/kg) served as a positive control. Liver function markers, oxidative stress indicators, and inflammatory markers were assessed. Transcriptomic and network pharmacology analyses identified key genes and pathways, validated by reverse transcription quantitative polymerase chain reaction (RT-qPCR) and Western blotting. Results: UPLC-Q-Orbitrap-MS identified 81 CGE compounds, mainly including terpenoids, flavonoids, and phenylpropanoids. CGE significantly ameliorated liver injury by reducing alanine aminotransferase (ALT), aspartate aminotransferase (AST), and alkaline phosphatase (ALP) levels and enhancing antioxidative markers such as total antioxidant capacity (T-AOC) and total superoxide dismutase (T-SOD) while lowering hepatic malondialdehyde (MDA) levels. Inflammation was mitigated through reduced levels of Tumor Necrosis Factor Alpha (TNF-α), Interleukin-1 Beta (IL-1β), and C-X-C Motif Chemokine Ligand 10 (CXCL-10). Transcriptomic and network pharmacology analysis revealed seven key antioxidant-related genes, including *HMOX1*, *RSAD2*, *BCL6*, *CDKN1A*, *THBD*, *SLC2A4*, and *TGFβ3*, validated by RT-qPCR. CGE activated the P21/Nuclear Factor Erythroid 2-Related Factor 2 (Nrf2)/Heme Oxygenase-1 (HO-1) signaling axis, increasing P21, Nrf2, and HO-1 protein levels while suppressing Kelch-like ECH-associated Protein 1 (Keap1) expression. Conclusions: CGE mitigates oxidative stress and liver injury by activating the P21/Nrf2/HO-1 pathway and regulating antioxidant genes. Its hepatoprotective effects and multi-target mechanisms highlight CGE’s potential as a promising therapeutic candidate for ALD treatment.

## 1. Introduction

Alcoholic liver disease (ALD) is a group of liver disorders caused by chronic excessive alcohol consumption, encompassing stages such as simple steatosis, alcoholic hepatitis, and alcoholic cirrhosis. ALD leads to roughly 25% of cirrhosis-related deaths and 20% of liver cancer-related fatalities worldwide, making it one of the primary contributors to liver disease-related mortality [1,2,3]. While the underlying mechanisms of ALD are intricate and involve multiple factors, including oxidative stress, inflammatory responses, and lipid metabolism disorders, oxidative stress is widely recognized as a key driver of its onset and progression [4]. Reactive oxygen species (ROS) generated during alcohol metabolism trigger lipid peroxidation and harm to proteins and DNA, leading to mitochondrial dysfunction, the apoptosis of hepatocytes, and the activation of inflammatory cascades. Therefore, mitigating oxidative stress is considered a critical strategy to slow the progression of ALD [5].

Currently, treatments for ALD primarily focus on alcohol abstinence, nutritional support, and symptomatic management. However, there is a lack of specific drugs, and the effectiveness of existing therapies is limited. Moreover, some patients exhibit poor tolerance to conventional treatments [6]. Natural products, with their multi-target effects and low toxicity, are considered promising candidates for ALD treatment [7]. Research has demonstrated that natural compounds can mitigate alcohol-induced oxidative stress by activating the Nuclear Factor Erythroid 2-Related Factor 2 (Nrf2) signaling pathway, which upregulates antioxidant enzymes like Heme Oxygenase-1 (HO-1) and helps eliminate excess ROS [8]. For example, extracts from Lycium ruthenicum and scutellarin have been shown to mitigate alcohol-induced hepatic damage through the Nrf2/HO-1 pathway [9,10]. Nevertheless, it remains necessary to identify more natural compounds with antioxidant potential and elucidate their mechanisms of action.

*Cichorium glandulosum* Boiss. et Huet., a member of the Asteraceae family, refers to the plant’s dried aerial parts or roots, which is classified as both a medicinal and edible herb [11]. Traditional Chinese medicines developed with *Cichorium glandulosum* as a main ingredient, such as Qingre Kasan Granules and Hugan Buzure Granules, have demonstrated significant hepatoprotective effects in clinical practice [12]. *Cichorium glandulosum* is rich in bioactive compounds, including terpenoids, flavonoids, and phenylpropanoids [13,14]. Its ethyl acetate extract (CGE) contains sesquiterpenoids as the major active components and exhibits antioxidant, anti-inflammatory, antibacterial, and hepatoprotective activities [15,16,17]. Previous studies from our research group have shown that sesquiterpenoids such as lactucopicrin and lactucin, isolated from CGE, significantly upregulate Nrf2 expression and alleviate oxidative stress in high-fat-diet-induced mice [18]. These observations imply that CGE might alleviate oxidative stress caused by ALD through the activation of the Nrf2 pathway, although the exact mechanisms responsible for its effects are not completely elucidated.

Multi-omics technologies have been widely applied in natural product research, particularly the integration of transcriptomics and network pharmacology. This approach enables the rapid prediction of potential drug targets and mechanisms of action, thereby enhancing the efficiency of natural product development [19,20]. For example, Cai et al. employed transcriptomics combined with network pharmacology to identify the bioactive compounds and anti-fibrotic molecules of the Yinchenhao decoction [21]. Our previous studies using transcriptomics and cell-based experiments demonstrated that sesquiterpenoids such as lactucopicrin and lactucin from *Cichorium glandulosum* improve lipid droplet accumulation by regulating fatty acid oxidation pathways in steatotic HepG2 cells [22]. These discoveries offer a valuable understanding of the multi-target mechanisms via which CGE alleviates ALD.

Based on this, the present study utilized Ultra-Performance Liquid Chromatography Quadrupole Orbitrap Mass Spectrometry (UPLC-Q-Orbitrap-MS) to characterize the chemical composition of CGE and assessed its antioxidative stress effects in an alcohol-induced C57BL/6J mouse model of ALD. Transcriptomic analysis was performed to identify key genes associated with CGE’s protective effects on ALD, and network pharmacology was applied to pinpoint the core signaling pathways. Key targets were further verified using Reverse Transcription Quantitative Polymerase Chain Reaction (RT-qPCR) and Western blotting. This study not only provides scientific evidence for the application of CGE in the treatment of ALD but also offers theoretical support for the advancement of plant-derived antioxidants.

## 2. Materials and Methods

### 2.1. Preparation of the Ethyl Acetate Extract of Cichorium glandulosum (CGE)

*Cichorium glandulosum* was sourced from Moyu County, Hotan City, Xinjiang, and authenticated as the whole herb of the *Cichorium glandulosum* plant (Asteraceae family) by Professor Haiyan Xu, College of Pharmacy, Xinjiang Medical University.

The dried whole herb of *Cichorium glandulosum* was reflux-extracted with 80% ethanol (1:10, *w*/*v*) at 80 °C for 2 h. The process was carried out three times, and the combined extracts were filtered, concentrated, and dried to yield the ethanol extract. The ethanol extract was reconstituted in purified water (1:10, *w*/*v*) with ultrasonication to ensure complete dissolution. The resulting solution was sequentially extracted three times with petroleum ether and then with ethyl acetate. The ethyl acetate extracts were pooled, and the solvent was evaporated under reduced pressure. The final CGE was dried and stored in a sealed container at 4 °C under light-protected conditions.

### 2.2. UPLC-Q-Orbitrap-MS Analysis

#### 2.2.1. Sample Preparation

The sample was pulverized in liquid nitrogen and transferred into a centrifuge tube. A water solution containing 4 μg/mL mixed internal standards was added, followed by 1 min vortexing. After being pre-cooled to −40 °C, the sample was ground for 2 min, followed by sonication in an ice bath for 60 min. It was then centrifuged at 14,000 rpm for 10 min at 4 °C. A 200 μL portion of the supernatant was transferred to an LC-MS vial with a liner for further analysis.

#### 2.2.2. Chromatographic and Mass Spectrometric Conditions

The components of CGE were analyzed using a UPLC-Q-Orbitrap-MS system. Chromatographic separation was carried out on an ACQUITY UPLC I-Class HF system (Waters, Milford, MA, USA) equipped with an ACQUITY UPLC HSS T3 column (100 mm × 2.1 mm, 1.8 μm, Waters). The column temperature was maintained at 45 °C, with a mobile phase comprising water containing 0.1% formic acid (Phase A) and acetonitrile (Phase B). The flow rate was 0.35 mL/min, and the injection volume was 5 μL. UV detection was performed using a Photodiode Array (PDA) detector with a scanning range of 210–400 nm. The gradient elution conditions are summarized in Table S1.

Mass spectrometric analysis was performed using a Thermo Orbitrap QE high-resolution LC-MS system equipped with a High-Energy Collision Dissociation (HESI) source. Data acquisition was conducted using Data-Dependent Acquisition (DDA). The mass spectrometry parameters are listed in Table S2.

#### 2.2.3. Qualitative Analysis of Compounds

Data processing, including baseline correction, peak identification, integration, retention time alignment, and normalization, was performed using Progenesis QI v3.0 software (Nonlinear Dynamics, Newcastle, UK). Compounds were characterized using precise mass, secondary fragment information, and isotope distribution characteristics, with annotations referencing the HerbDB and LuMet-TCM databases.

### 2.3. Animal Experiment

#### 2.3.1. Experimental Design

Fifty male SPF-grade C57BL/6J mice (6 weeks old, 20 ± 2 g) were housed in the SPF animal facility of the Xinjiang Medical University Animal Experiment Center (SYXK(Xin)2023-0004). Mice were maintained under a controlled environment (25 ± 1 °C, 55–65% relative humidity) with a 12 h light/dark cycle and unrestricted food and water supply. Following a 2-week acclimatization period, the mice were randomly divided into five groups (n = 10/group): control (Con), model (Mod), low-dose CGE (CGE-L), high-dose CGE (CGE-H), and positive drug Silibinin (SIL) groups. Ethical approval was granted by the Ethics Committee of Experimental Animals, Xinjiang Medical University (Approval No. IACC-JT-20230831-31).

#### 2.3.2. Establishment of Alcohol-Induced Liver Injury Model

After acclimation, all mice except the control group were gavaged daily with 56% Baijiu (10 mL/kg; Beijing Red Star Co., Ltd., Beijing, China, Lot: 20190929). Two hours later, the CGE-L and CGE-H groups were gavaged with CGE solutions (10 mg/mL and 20 mg/mL), and the SIL group was gavaged with a 10 mg/mL silibinin solution, all at 10 mL/kg. The control and model groups received an equivalent volume of solvent. Treatments were administered daily for six weeks.

CGE solutions were prepared by dissolving CGE (100 mg or 200 mg) in 10 mL of 1% Tween-80 solution (Beijing Solarbio, Beijing, China, CAT: T8360) with ultrasonic treatment to yield 10 mg/mL and 20 mg/mL concentrations. Similarly, silibinin solution (10 mg/mL) was prepared by dissolving 100 mg of silibinin (Tianjin Tasly-Synthe Pharmaceutical Co., Ltd., Tianjin, China, Lot: 350705027) in 10 mL of 1% Tween-80 solution. The CGE dosage in this study was referenced from the dosage used by Han et al. [16].

#### 2.3.3. Collection of Animal Samples

Blood samples were left at room temperature for 1 h, centrifuged at 4 °C (10 min, 3000 rpm) to isolate serum, and stored at −80 °C. Liver tissues were fixed in 4% paraformaldehyde for histology or frozen at −80 °C for biochemical analysis.

#### 2.3.4. Measurement of Body Weight and Liver Index

Body weights and liver weights were recorded. The liver index was calculated using the formula:

Liver Index (g/g) = Liver Weight (g)/Mouse Body Weight (g) × 100%.

#### 2.3.5. Histological Observation of Liver Tissue Pathology by H&E Staining

Liver tissues fixed were dehydrated in an ethanol gradient, embedded in paraffin, sectioned, mounted, and stained with hematoxylin and eosin (H&E). Liver tissues were observed under the microscope (Eclipse C-E, Nikon, Kanagawa, Japan).

#### 2.3.6. Measurement of Hepatic Function and Inflammatory Indicators

Hepatic function markers alanine aminotransferase (ALT), aspartate aminotransferase (AST), and alkaline phosphatase (ALP) were measured using biochemical assay kits (Nanjing Jiancheng Bioengineering Institute, Nanjing, China, CAT: C009-2-1, C010-2-1, A059-2). Levels of tumor necrosis factor-alpha (TNF-α), interleukin-1 beta (IL-1β), and C-X-C motif chemokine ligand 10 (CXCL-10) in liver tissues were determined using ELISA kits (Shanghai Youxuan Biotechnology Co., Ltd., Shanghai, China, CAT: YX-201407M, YX-091203M, YX-091610M).

#### 2.3.7. Measurement of Oxidative Stress Markers

Serum total antioxidant capacity (T-AOC), total superoxide dismutase (T-SOD), and liver malondialdehyde (MDA) levels were quantified using biochemical assay kits (Elabscience Biotechnology Co., Ltd., Wuhan, China, CAT: E-BC-K136-M, E-BC-K020-M, E-BC-K025-M).

### 2.4. Transcriptomic Experiments and Analysis

#### 2.4.1. Transcriptome Sequencing

Three mice were randomly selected from the control, model, and CGE-H groups as biological replicates. RNA was purified from liver tissues using the TRNzol Universal RNA Kit (TIANGEN, CAT:DP424). RNA quality was assessed with a NanoDrop ND-1000 spectrophotometer and agarose gel electrophoresis (Bio-Rad PowerPac Universal, Bio-Rad, Hercules, CA, USA). RNA libraries were constructed with the Hieff NGS^®^ Ultima Dual-mode RNA Library Prep Kit (Premixed version), and library quality was verified with the Agilent 4200 TapeStation. High-throughput sequencing was performed on an Illumina platform.

#### 2.4.2. RNA-Seq Data Analysis

Raw sequencing data were preprocessed using Fastp software (v0.23.0). The resulting clean reads were aligned to the reference genome using HISAT2 (v2.1.0). Gene expression levels were calculated using StringTie (v2.2.1) and normalized to Transcripts Per Kilobase Million (TPM) to account for differences in gene length and sequencing depth. Differentially expressed genes (DEGs) were determined using criteria of |log_2_(FoldChange)| > 1 and *p* < 0.05.

#### 2.4.3. Screening for Potential Targets of CGE in Alleviating ALD-Induced Oxidative Stress

Oxidative stress-related gene data were retrieved from the GeneCards database. Venn diagram analysis was performed using the Venn tool to identify intersecting genes among oxidative stress-related genes, DEGs from the CGE-treated group, and DEGs from the model group. These intersecting genes were recognized as candidate targets for CGE in ameliorating ALD-induced oxidative stress.

#### 2.4.4. Gene Ontology (GO) and Kyoto Encyclopedia of Genes and Genomes (KEGG) Analysis

GO functional enrichment and KEGG pathway enrichment analyses were conducted using the DAVID database.

#### 2.4.5. Identification of Key Genes Involved in CGE’s Improvement of Oxidative Stress in ALD Mice

The intersecting genes from Section 2.4.3 were uploaded to the STRING 12.0 database to construct a Protein-Protein Interaction (PPI) network. Key genes in the network were identified using the cytoHubba plugin in Cytoscape 3.8.2. The GeneMANIA database was also used to predict interactions with the *NFE2L2* gene [23].

### 2.5. Network Pharmacology Analysis

#### 2.5.1. Target Prediction for CGE Chemical Components

A total of 81 potentially active compounds in CGE, identified through UPLC-Q-Orbitrap-MS, were subjected to network pharmacology analysis. The SDF files of these compounds were obtained from the PubChem database and uploaded to the SwissTargetPrediction and PharmMapper platforms for target prediction. Predicted targets were normalized and mapped to official gene symbols using the UniProt database.

#### 2.5.2. Target Screening for CGE in Treating ALD-Induced Oxidative Stress

Disease-related genes were retrieved by searching the keyword “Alcoholic Liver Disease” in GeneCards (score > 1) and DisGeNET (score > 0.1). Similarly, oxidative stress-related genes were retrieved by searching the keyword “Oxidative Stress” in GeneCards (score > 1). The Venn diagram tool was applied to identify intersecting genes among CGE compound targets, ALD targets, and oxidative stress targets. These intersecting genes were considered potential targets of CGE for ameliorating ALD-induced oxidative stress.

#### 2.5.3. GO and KEGG Analysis

GO terms and KEGG pathways were conducted using the DAVID database.

#### 2.5.4. Integrated Transcriptomic and Network Pharmacology Analysis

To further elucidate the antioxidative mechanisms of CGE in ALD treatment, KEGG pathway enrichment was performed on key genes identified via Cytoscape. The Venn diagram tool was employed to identify overlapping KEGG pathways derived from network pharmacology and transcriptomic analyses. Genes involved in the intersecting key pathways were subsequently validated through qRT-PCR experiments.

### 2.6. Validation of Key Genes Regulated by CGE in Alleviating ALD-Induced Oxidative Stress Using RT-qPCR

Reverse transcription was carried out with the PrimeScript RT Reagent Kit (Takara Bio, CAT: RR047A, Shizuoka, Japan) to eliminate genomic DNA contamination. RT-qPCR was conducted with the TB Green Premix Ex Taq II Kit (Takara, CAT: RR047A), Sangon Biotech Co., Ltd. (Shanghai, China), with custom-designed and synthesized primers for target genes. Detailed primer sequences are provided in Table S3.

### 2.7. Western Blotting to Detect Protein Targets in the P21/Nrf2/HO-1 Signaling Axis

Liver tissues were homogenized using a tissue homogenizer in RIPA lysis buffer. Homogenates were centrifuged, proteins quantified with a BCA kit (Beijing Solarbio; CAT: PC0020), and adjusted to uniform concentrations for analysis.

Proteins were mixed with Sodium Dodecyl Sulfate-Polyacrylamide Gel Electrophoresis (SDS-PAGE) loading buffer (Beijing Solarbio Science & Technology Co., Ltd., Beijing, China; CAT: P1040), denatured in a 95 °C water bath for 5 min, and rapidly cooled on ice. Proteins underwent denaturation, were resolved by SDS-PAGE, and subsequently transferred onto Polyvinylidene Fluoride (PVDF) membranes. The membranes were then blocked using 5% skim milk.

Primary antibodies against Nrf2 (Wuhan Sanying Biotechnology Co., Ltd. Wuhan, China; CAT: 80593-1-RR), HO-1, Keap1, and P21 (Cell Signaling Technology, Danvers, MA, US, CAT: 43966, 8047, 37543), and β-actin (Affinity Biosciences, Changzhou, China, CAT: AF7018) were applied and incubated overnight at 4 °C. The following day, secondary antibodies were applied to the membranes for incubation (Affinity Biosciences, CAT: S0001), and protein bands were visualized. Signal intensity was analyzed using an imaging system to assess protein expression levels.

### 2.8. Statistical Analysis

Results were shown as mean ± SD, with protein bands analyzed in ImageJ (v2.14.0). Statistical tests used GraphPad Prism 9.0, and one-way ANOVA determined significance at *p* < 0.05.

## 3. Results

### 3.1. Identification of CGE Components by UPLC-Q-Orbitrap-MS

**Figure 1:**
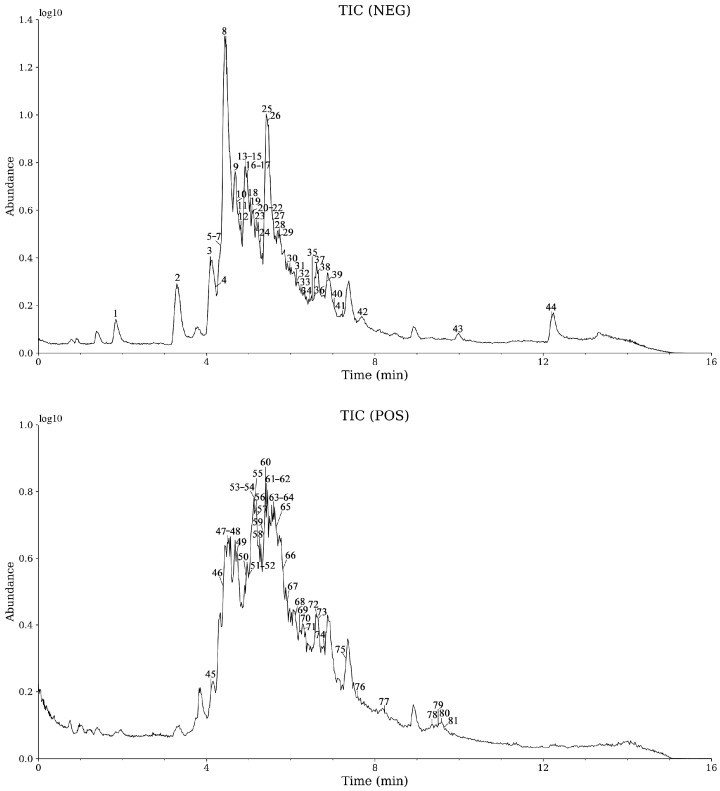
Total ion chromatogram (TIC).

A total of 81 compounds in CGE were identified using UPLC-Q-Orbitrap-MS, with the ion chromatogram depicted in Figure 1. The names and classifications of these compounds were standardized using the PubChem database. Detailed information, including compound names, retention times, fragment ions, and classifications, is provided in Table S4.

#### 3.1.1. Terpenoids

A total of 23 terpenoids were identified, including Isololiolide, Barrelin, Britannilactone, Chinensiolide B, 4α,5α-epoxy-6α-hydroxyamorphan-12-oic acid, Pseudolaric Acid C, Paeonilactone B, 2,3,11,13-Tetrahydrohelenalin, Nardosinone, 1-Deoxyeucommiol, Santonin, Austricine, Lactucin, 11β,13-dihydroreynosin, 11,13-dihydrolactucin, L-α-Hydroxyarbusculin A, Blumenol C, 8-deoxylactucin, Coronopolin, 5-(2,5-dihydrofuran-3-yl)-2-methylpent-3-en-2-ol, 11β,13-Dihydrolactucopicrin, Lactucopicrin, and Olibanumol B.

**Figure 2:**
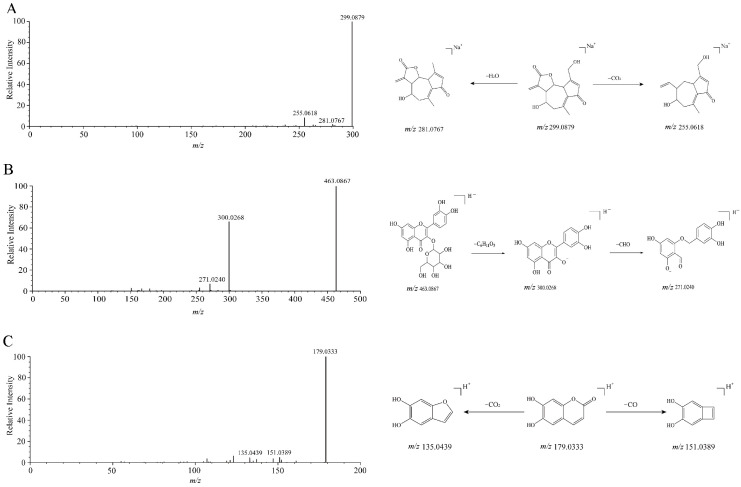
Proposed fragmentation pathways of representative compounds. (**A**) Secondary mass spectra of lactucin and its proposed fragmentation pathways. (**B**) Secondary mass spectra of isoquercitrin and its proposed fragmentation pathways. (**C**) Secondary mass spectra of fraxin and its proposed fragmentation pathways.

Taking Lactucin as an example, its quasi-molecular ion peak in positive ion mode was identified as [M + Na]^+^ *m*/*z* 299.0877, with a predicted molecular formula of C_15_H_16_O_5_. Characteristic fragment ions included [M − H_2_O]^+^ *m*/*z* 281.0767 and [M − CO_2_]^+^ *m*/*z* 255.0618. Based on the HerbDB database, the fragmentation pattern of Lactucin was highly consistent with the characteristic fragments of this compound, leading to its identification as Lactucin. The MS/MS spectrum and the proposed fragmentation pathway are shown in Figure 2A.

#### 3.1.2. Flavonoids

A total of 13 flavonoids were identified, including Quercetin 3-O-β-D-glucofuranoside, Brassicin, Isoquercitrin, Quercitrin, Kaempferol 3-O-β-D-glucuronide, Isorhamnetin-3-O-β-D-glucoside, Trilobatin, Ononin, Quercetin, Morin, Kaempferol, Isotrifoliol, and Calycosin.

Taking Isoquercitrin as an example, its quasi-molecular ion peak in negative ion mode was identified as [M − H]^−^ *m*/*z* 463.0867, with a predicted molecular formula of C_21_H_20_O_12_. Characteristic fragment ions included [M − C_6_H_11_O_5_]^−^ *m*/*z* 300.0268 and [M − C_6_H_11_O_5_-O_2_]^−^ *m*/*z* 271.0240. Based on the LuMet-TCM database, the fragmentation pattern of Isoquercitrin was highly consistent with the characteristic fragments of this compound, leading to its identification as Isoquercitrin. The MS/MS spectrum and the proposed fragmentation pathway are shown in Figure 2B.

#### 3.1.3. Phenylpropanoids

A total of 14 phenylpropanoids were identified, including Dihydrocaffeic acid, Esculetin, 3-Feruloylquinic acid, Isorhapontigenin, (*E*)-p-Coumaric acid, Isoferulic acid, 3,4-Dimethoxycinnamic acid, Syringaresinol, Isochlorogenic acid C, Scopoletin, 3,4,5-Trimethoxyhydrocinnamic acid, Sinapaldehyde, Vaccaxanthone, and Ethyl Caffeic acid.

Taking Esculetin as an example, its quasi-molecular ion peak in negative ion mode was identified as [M − H]^−^ *m*/*z* 179.0333, with a predicted molecular formula of C_9_H_6_O_4_. Characteristic fragment ions included [M − CO]^−^ *m*/*z* 151.0389 and [M − CO_2_]^−^ *m*/*z* 135.0439. Based on the LuMet-TCM database, the fragmentation pattern of Esculetin was highly consistent with the characteristic fragments of this compound, leading to its identification as Esculetin. The MS/MS spectrum and the proposed fragmentation pathway are shown in Figure 2C.

#### 3.1.4. Other Compounds

The remaining components included the following: 9 phenolic compounds, namely Gallic acid, Syringaldehyde, Feroxidin, Ethyl gallate, Ellagic acid, Isovanillin, 3,4-Dihydroxybenzaldehyde, 3-Hydroxybenzaldehyde, and Salicyl alcohol; seven organic acids and derivatives, namely Gentisic acid, Phthalic acid, (2*R*)-2-butoxybutanedioic acid, 3-(3-Hydroxyphenyl)propanoic acid, Ferulic acid, 6-Oxooctanoic acid, and Caffeic acid; four fatty acids, namely 4-hydroxy-2-hexenoic acid, Suberic acid, Traumatic acid, and 3-Hydroxypalmitic acid; three carboxylic acids and derivatives, namely 3,5-Dihydroxybenzoic acid, 3-O-p-coumaroylquinic acid, and Ethyl 3,4-dihydroxybenzoate; seven quinones, namely Aloeresin D, Torachrysone-8-O-β-D-glucoside, and Emodin; two alkaloids, namely Methyl 5-hydroxypyridine-2-carboxylate and Piperine; two sphingolipids, namely Dehydrophytosphingosine and Phytosphingosine; and one apocarotenoid, namely Sec-hydroxyaeginetic acid.

### 3.2. CGE Alleviates Liver Injury and Enhances Antioxidative Stress Levels in ALD Mice

**Figure 3:**
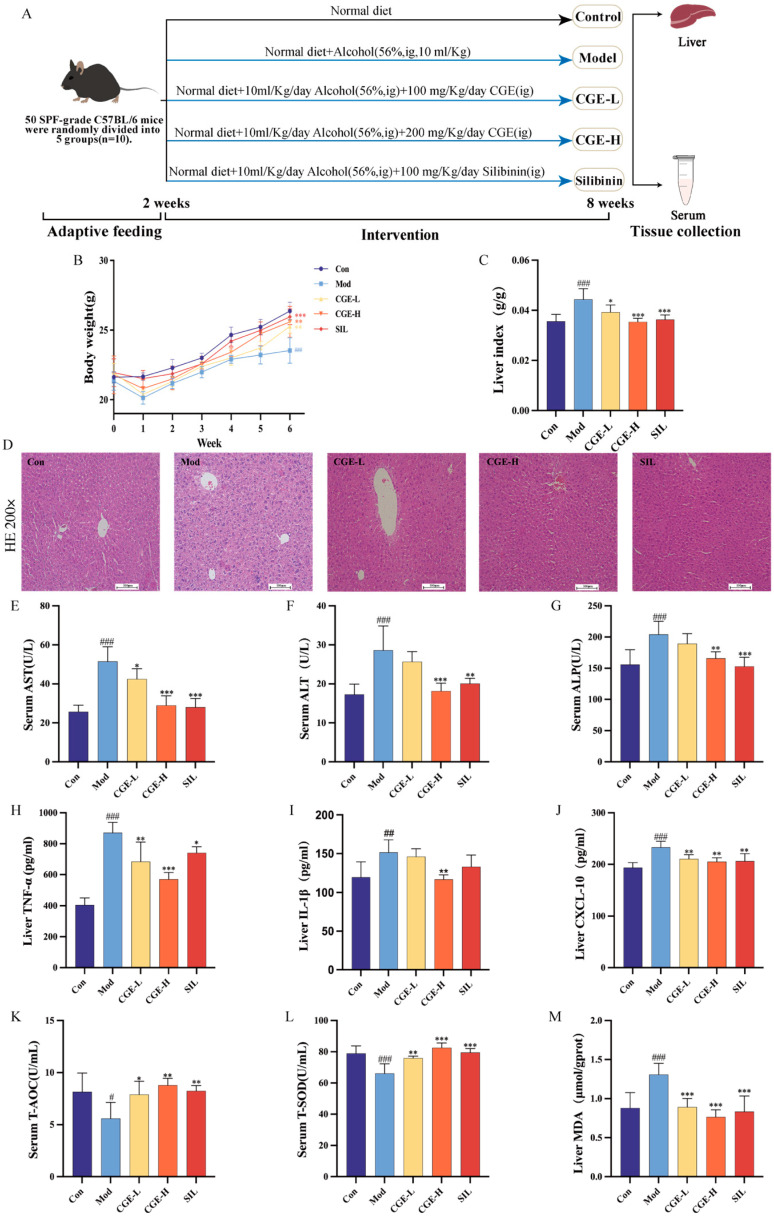
CGE alleviates alcohol-induced liver injury and enhances antioxidative stress levels in mice. (**A**). Animal experiment design. (**B**). The effects of CGE on the body weight of ALD mice. (**C**). The effects of CGE on the liver index of ALD mice. (**D**). Liver tissue H&E staining (200×, scale bar = 100 μm). (**E**). The effects of CGE on serum AST levels in ALD mice. (**F**). The effects of CGE on serum ALT levels in ALD mice. (**G**). The effects of CGE on serum ALP levels in ALD mice. (**H**). The effects of CGE on hepatic TNF-α levels in ALD mice. (**I**). The effects of CGE on hepatic IL-1β levels in ALD mice. (**J**). The effects of CGE on hepatic CXCL-10 levels in ALD mice. (**K**). The effects of CGE on serum T-AOC levels in ALD mice. (**L**). The effects of CGE on serum T-SOD levels in ALD mice. (**M**). The effects of CGE on hepatic MDA levels in ALD mice. Data are presented as mean ± SD (n = 6). Relative to the control group, ^#^ *p* < 0.05, ^##^ *p* < 0.01, and ^###^ *p* < 0.001; relative to the model group, * *p* < 0.05, ** *p* < 0.01, and *** *p* < 0.001.

The component identification results revealed that CGE comprises an array of potentially bioactive substances, primarily including terpenoids, flavonoids, and phenylpropanoids. To investigate the defensive and antioxidative effects of CGE against ALD in animal models, a murine model of hepatic damage caused by 56% ethanol was established (Figure 3A), with SIL used as a positive control for alcohol-triggered liver damage. By week 6, the model group showed considerably lower body weight when contrasted with the control group (*p* < 0.001). Administration of CGE-L (*p* < 0.01), CGE-H (*p* < 0.01), and SIL (*p* < 0.001) significantly alleviated the body weight loss (Figure 3B). The liver index of the model group demonstrated a notable elevation (*p* < 0.001). Treatment with CGE-L (*p* < 0.05), CGE-H (*p* < 0.001), and SIL (*p* < 0.001) markedly reduced the liver index (Figure 3C). The H&E staining of liver tissues (Figure 3D) revealed hepatocyte steatosis, ballooning degeneration, increased cell volume, irregular arrangement, and the invasion of inflammatory cells in the model group, which were alleviated in the CGE-H and SIL treatment groups. These findings indicate that CGE can mitigate ALD-induced weight loss, increased liver index, and pathological changes in liver tissues.

Further analysis of liver function markers (Figure 3E–G) revealed significant increases in serum levels of ALT, AST, and ALP in the model group (*p* < 0.001). Compared to the model group, CGE-L significantly reduced AST levels (*p* < 0.05), while CGE-H and SIL significantly reduced AST (*p* < 0.001, *p* < 0.001), ALT (*p* < 0.001, *p* < 0.01), and ALP levels (*p* < 0.01, *p* < 0.001). Regarding pro-inflammatory cytokines (Figure 3H–J), the levels of TNF-α (*p* < 0.001), IL-1β (*p* < 0.01), and CXCL-10 (*p* < 0.001) were markedly elevated in the model group when compared to the control group. After treatment, CGE-L significantly reduced TNF-α (*p* < 0.01) and CXCL-10 (*p* < 0.01) levels. The CGE-H group significantly reduced TNF-α (*p* < 0.001), IL-1β (*p* < 0.01), and CXCL-10 (*p* < 0.01) levels. The SIL group significantly reduced TNF-α (*p* < 0.05) and CXCL-10 (*p* < 0.01) levels. In Figure 3K–M, the model group exhibited significantly reduced levels of serum T-AOC and T-SOD as opposed to the control group (*p* < 0.05 and *p* < 0.001, respectively). Conversely, hepatic levels of MDA showed a substantial increase in the model group (*p* < 0.001). After treatment, T-AOC and T-SOD levels were significantly elevated in the CGE-L (*p* < 0.05, *p* < 0.01), CGE-H (*p* < 0.01, *p* < 0.001), and SIL (*p* < 0.01, *p* < 0.001) groups, while MDA levels were notably reduced (*p* < 0.001, *p* < 0.001, *p* < 0.001). These findings indicate that CGE reduces liver injury and improves antioxidative stress levels in ALD mice, with the high-dose group exhibiting greater efficacy compared to the low-dose group.

### 3.3. Transcriptomic Analysis of CGE’s Antioxidative Effects in ALD Mice

**Figure 4:**
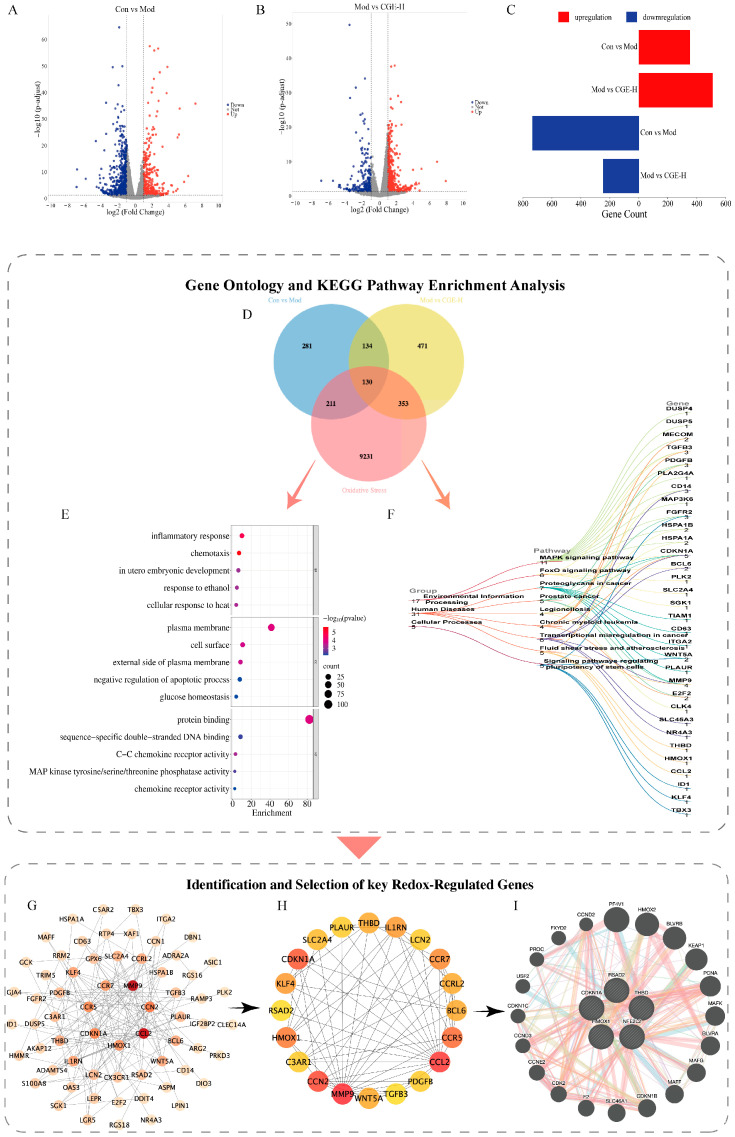
Differential gene screening, enrichment analysis, and identification of key antioxidative genes from transcriptomics. (**A**). Volcano plot for Con vs. Mod. (**B**). Volcano plot for Mod vs. CGE-H. (**C**). Number of markedly upregulated and downregulated genes within the Con vs. Mod and Mod vs. CGE-H groups. (**D**). Venn diagram of DEGs from Con vs. Mod, DEGs from Mod vs. CGE-H, and oxidative stress-related genes. (**E**). Bubble chart of GO analysis for CGE’s potential antioxidative stress-related genes. (**F**). Sankey diagram of KEGG pathways for CGE’s potential antioxidative stress genes. (**G**). PPI network of 130 genes associated with antioxidative stress under CGE regulation. (**H**). Identification of the top 20 key genes using the Degree algorithm in the cytoHubba plugin. Lines between circles represent interactions between genes, and circle colors ranging from red to yellow indicate interaction strength from high to low. (**I**). Using the GeneMANIA database, four genes interacting with *NFE2L2* were identified from the 20 key genes.

In the transcriptomic analysis, we explored the molecular mechanisms of CGE’s antioxidative effects by examining the variations in gene expression across the various treatment groups in the ALD mouse model. In the model group, 1088 DEGs were detected relative to the normal group, including 353 upregulated and 735 downregulated genes. In the CGE-H group, relative to the model group, a total of 756 DEGs were detected, comprising 509 genes with significant upregulation and 247 with significant downregulation. Figure 4A–C illustrate the volcano plot of these DEGs as well as the number of upregulated and downregulated genes.

From the GeneCards database, 9925 oxidative stress-related genes have been discovered. A total of 130 overlapping genes were discovered among the DEGs from the Mod vs. Con group, the DEGs from the CGE-H vs. Mod group, and oxidative stress-related genes. These genes represent potential target genes for CGE in treating oxidative stress caused by ALD (Figure 4D). These 130 target genes were further analyzed through GO and KEGG enrichment analyses. GO analysis revealed significant enrichment in 69 biological process (BP), 61 cellular component (CC), and 14 molecular function (MF). Figure 4E highlights the top five enriched terms in each category. In the BP category, the CGE antioxidant potential targets were highly concentrated in biological processes including inflammatory response, chemotaxis, embryonic development in the uterus, response to ethanol, and response to heat. In the CC category, these targets were predominantly localized to the plasma membrane, cell surface, and extracellular side of the plasma membrane, as well as being associated with processes such as the negative regulation of apoptosis and glucose homeostasis. For MF, the targets were enriched in molecular functions including protein binding, sequence-specific double-stranded DNA binding, C-C chemokine receptor activity, MAP kinase tyrosine/serine/threonine phosphatase activity, and chemokine receptor activity. KEGG pathway analysis further showed that 130 potential target genes were significantly enriched in nine pathways (Figure 4F), including the Mitogen-Activated Protein Kinase (MAPK) and Forkhead Box O (FOXO) signaling pathways.

The PPI network for 130 potential target genes of CGE was constructed using the STRING database (Figure 4G). With the cytoHubba plugin in Cytoscape 3.8.2 (Figure 4H), the Degree algorithm identified 20 pivotal genes, namely *MMP9*, *CCL2*, *CCN2*, *CDKN1A*, *CCR5*, *HMOX1*, *CCR7*, *KLF4*, *IL1RN*, *THBD*, *WNT5A*, *BCL6*, *CCRL2*, *SLC2A4*, *PDGFB*, *LCN2*, *PLAUR*, *C3AR1*, *RSAD2*, and *TGFB3*. The deeper the color, the higher the Degree value and the more significant the gene in the network.

Given the critical regulatory role of *NFE2L2* (the gene encoding Nrf2) in alleviating oxidative stress in ALD, the study further utilized the GeneMANIA database to analyze the key gene network regulated by CGE, aiming to identify genes directly or indirectly interacting with *NFE2L2* (Figure 4I). Four genes were identified to be associated with *NFE2L2*, including *HMOX1*, *CDKN1A*, *RSAD2*, and *THBD*. These genes may collectively participate in the antioxidative stress mechanism of CGE.

### 3.4. Network Pharmacology Analysis of CGE’s Antioxidative Effects in ALD Mice

**Figure 5:**
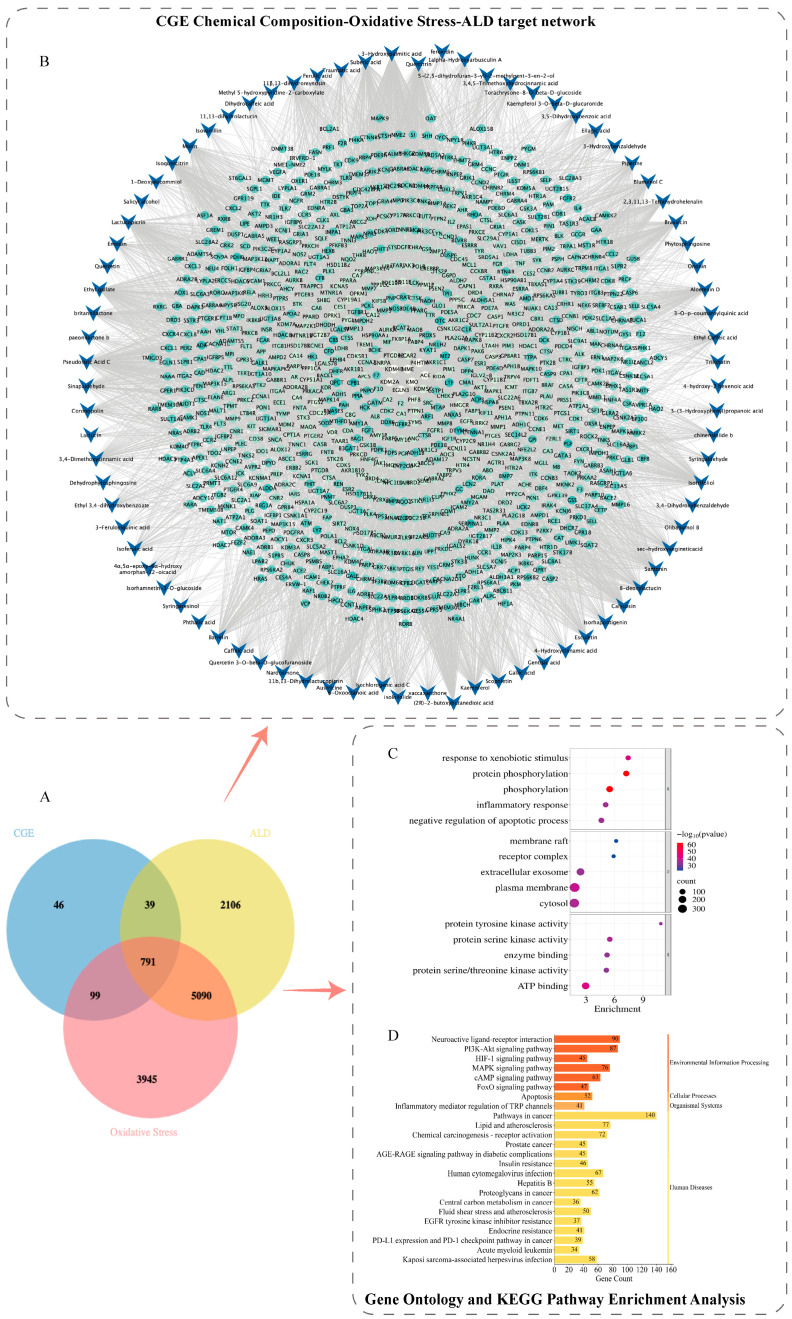
Mechanisms of CGE against ALD-induced oxidative damage identified via network pharmacology analysis. (**A**). Venn diagram of oxidative stress-related genes, CGE differential genes, and ALD genes, identifying potential gene targets of CGE against ALD-induced oxidative stress. (**B**). CGE compound–target network diagram. (**C**). GO analysis for potential genes of CGE against ALD-induced oxidative stress. (**D**). KEGG analysis for potential genes of CGE against ALD-induced oxidative stress.

To elucidate the antioxidative stress mechanisms of CGE, this study further incorporated network pharmacology approaches to analyze the key molecular networks and signaling pathways regulated by CGE, building upon the transcriptomics findings. A total of 8026 genes associated with ALD were gathered from the GeneCards and DisGeNET databases. Using the Venn tool, 791 overlapping targets were identified among the predicted targets of CGE active components, ALD targets, and oxidative stress targets, representing possible targets of CGE for combating ALD-induced oxidative stress (Figure 5A). A CGE compound–ALD–oxidative stress target network was established through Cytoscape.3.8.2 to investigate the connections linking the 81 active compounds with 791 overlapping targets. The CGE compound–ALD–oxidative stress target network comprised 1056 nodes and 5464 edges. Blue “V”-shaped nodes represent active components in CGE, while green circular nodes represent potential targets (Figure 5B).

GO and KEGG pathway analyses were performed for 791 potential antioxidant stress targets of CGE. The GO enrichment results (Figure 5C) revealed that these targets were associated with 122 BP, 20 CC, and 312 MF. The top five enriched terms in each category were highlighted. For BP, the targets were significantly clustered in processes including the negative modulation of apoptotic processes, inflammatory responses, peptidyl-tyrosine autophosphorylation, protein phosphorylation, and responses to xenobiotic stimuli. In the CC category, the targets were predominantly localized in the cytoplasm, plasma membrane, extracellular exosomes, receptor complexes, and membrane rafts. Regarding MF, the targets exhibited significant enrichment in molecular functions, including ATP binding, protein serine/threonine kinase activity, enzyme binding, and protein tyrosine kinase activity. The KEGG revealed that the 791 targets were enriched in 208 pathways. Figure 5D highlights the top 25 pathways, with CGE’s potential targets for mitigating ALD-induced oxidative stress significantly enriched in the Phosphatidylinositol 3-Kinase/Akt Pathway (PI3K-Akt), Hypoxia Inducible Factor-1 (HIF-1), MAPK, Cyclic Adenosine Monophosphate (cAMP), and FOXO signaling pathways.

### 3.5. Integrated Transcriptomic and Network Pharmacology Analysis of CGE’s Antioxidative Mechanisms in ALD Mice

**Figure 6:**
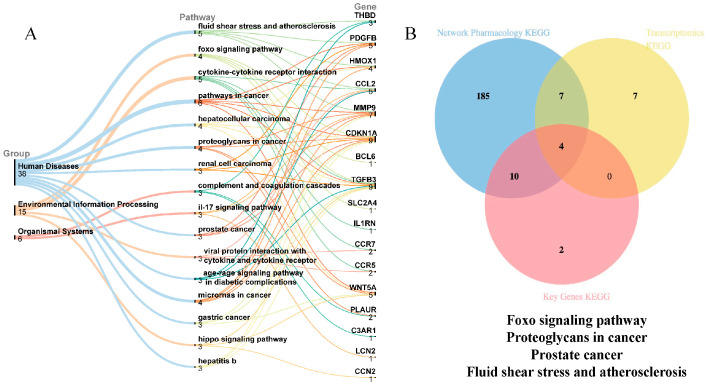
Integrated analysis of transcriptomics, network pharmacology, and key antioxidative genes. (**A**). KEGG Sankey diagram of the top 20 key genes identified using the Degree algorithm in the cytoHubba plugin. (**B**). Intersection pathways of network pharmacology, transcriptomics, and antioxidative core genes.

To further investigate how CGE mitigates oxidative damage in ALD, a comprehensive analysis integrating transcriptomics and network pharmacology was performed. The KEGG pathway analysis of the 20 key genes demonstrated their significant involvement in 16 pathways, including the FOXO, Interleukin 17 (IL-17), and Advanced Glycation End Products Receptor for Advanced Glycation End Products (AGE-RAGE) signaling pathways (Figure 6A). Using Venn analysis, the overlapping pathways between transcriptomics, network pharmacology, and the 20 key genes were identified as potential mechanisms of CGE against ALD-induced oxidative stress (Figure 6B). Four significantly enriched pathways were identified: the FOXO signaling pathway, proteoglycans in cancer, prostate cancer, and fluid shear stress and atherosclerosis. Among these, the FOXO signaling pathway was selected for further investigation due to its critical role in regulating cellular antioxidative stress and apoptosis, as well as its strong association with the pathological mechanisms of ALD [24]. Within this pathway, the key genes *CDKN1A*, *BCL6*, *TGFB3*, and *SLC2A4* were significantly enriched.

### 3.6. CGE Mitigates Oxidative Stress in ALD Mice by Activating the P21/Nrf2/HO-1 Signaling Axis and Regulating Multiple Genes

**Figure 7:**
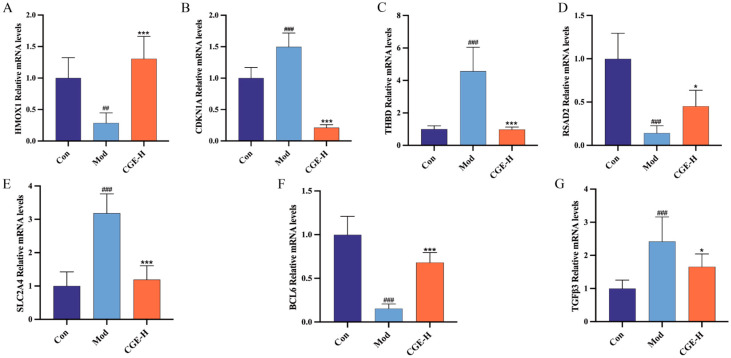
The validation of key antioxidative stress genes regulated by CGE. (**A**–**G**). Relative mRNA expression levels of *HMOX1*, *CDKN1A*, *THBD*, *RSAD*, *SLC2A4*, *BCL6*, *TGFβ3*. The results are presented as mean ± SD (n = 6). Relative to the control group, ^##^ *p* < 0.01, ^###^ *p* < 0.001; relative to the model group, * *p* < 0.05, *** *p* < 0.001.

To validate the seven core antioxidative stress genes identified through transcriptomic and network pharmacology analyses, the gene expression profiles were assessed, as illustrated in Figure 7A–G. In the model group, the expression levels of *HMOX1*, *RSAD2*, and *BCL6* were notably reduced, while those of *CDKN1A*, *THBD*, *SLC2A4*, and *TGFβ3* were significantly higher. After CGE-H treatment, these gene expression levels were significantly restored, consistent with the trends observed in the transcriptomic results.

**Figure 8:**
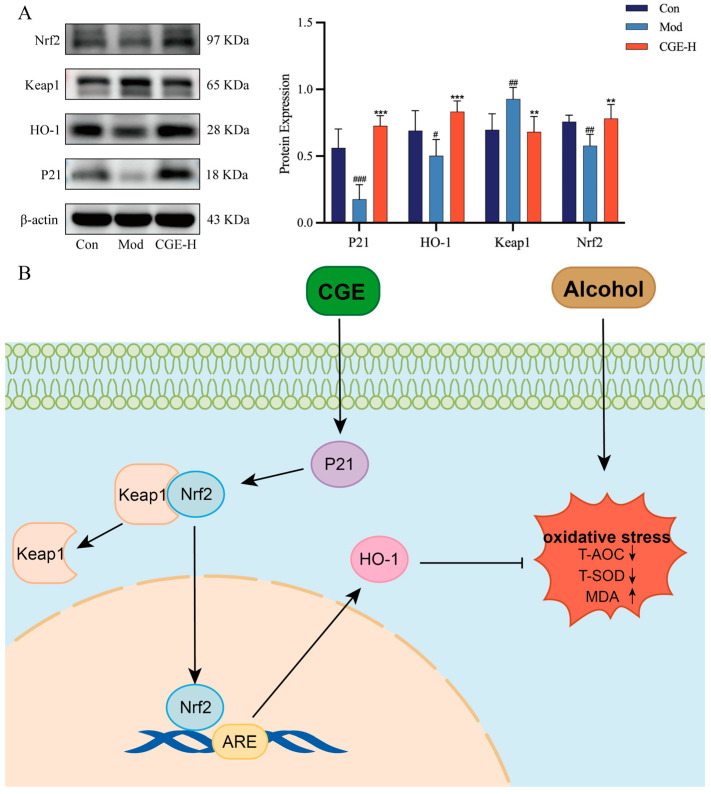
The process through which CGE mitigates oxidative stress in ALD mice involves the activation of the P21/Nrf2/HO-1 signaling pathway. (**A**). The analysis of protein expression levels and quantification for Nrf2, Keap1, HMOX1, and P21 in liver tissue. The results are presented as mean ± SD (n = 6). Statistical significance is indicated as follows: ^#^ *p* < 0.05, ^##^ *p* < 0.01, and ^###^ *p* < 0.001 compared to the control group; ** *p* < 0.01, and *** *p* < 0.001 compared to the model group. (**B**). CGE activates the P21/Nrf2/HO-1 pathway to reduce oxidative stress in ALD mice.

A literature review revealed that HO-1 (encoded by *HMOX1*) and P21 (encoded by *CDKN1A*) are closely associated with the Nrf2 and Kelch-like ECH-associated protein 1 (Keap1) proteins. Therefore, we performed Western blot analysis on P21, Nrf2, Keap1, and HO-1 to gain deeper insights into the antioxidative stress mechanism of CGE. Figure 8A,B illustrates that the Western blot analysis revealed that CGE-H treatment markedly increased P21, Nrf2, and HO-1 protein levels in contrast to the model group, while reducing Keap1 protein levels.

## 4. Discussion

Ethyl acetate extract of *Cichorium glandulosum* (CGE) has been shown to exhibit antioxidant, anti-inflammatory, antibacterial, and hepatoprotective bioactivities [15,16,17]. However, its potential role and mechanisms in mitigating ALD-associated oxidative stress remain unclear. This study, through transcriptomics, network pharmacology analyses, and systematic experimental validation, elucidated the mechanisms underlying CGE’s protective effects against ALD-induced oxidative stress. Furthermore, the findings provide a scientific foundation for the application of CGE in the development of multi-target therapeutic agents.

This study employed UPLC-Q-Orbitrap-MS technology to identify 81 chemical constituents in CGE, based on precise mass-to-charge ratios, secondary fragmentation patterns, and isotope distributions, which were cross-referenced with the HerbDB and LuMet-TCM databases. These compounds include 23 terpenoids, 13 flavonoids, 14 phenylpropanoids, 9 phenolic compounds, 7 organic acids and their derivatives, 4 fatty acids, 3 carboxylic acids and their derivatives, 3 quinones, 2 alkaloids, 2 sphingolipids, and 1 dehydrocarotenoid. Terpenoids, flavonoids, and phenylpropanoids are the most abundant classes of compounds in CGE. Studies have shown that these compounds, characterized by hydroxyl groups, conjugated structures, and unsaturated bonds in their molecular frameworks, exhibit antioxidant properties primarily through the scavenging of free radicals [25]. Previous research has demonstrated that compounds like lactucopicrin (a sesquiterpene), kaempferol (a flavonoid), and esculetin (a phenylpropanoid) possess both antioxidant and hepatoprotective effects [26,27,28], providing solid theoretical support for the antioxidant potential of CGE. However, the precise molecular targets and mechanisms underlying the actions of these compounds remain to be further explored.

This study employed an ALD mouse model induced by 56% ethanol and administered both low-dose and high-dose CGE interventions. The effects of CGE on alcohol-induced liver damage and oxidative stress were evaluated using various parameters, including body weight, liver index, liver function markers, inflammatory mediators, and oxidative stress indicators. The results showed that ethanol-induced ALD caused significant weight loss and an increased liver index in the mice, reflecting both nutritional deficiencies and liver injury [29]. The histopathological examination of liver tissues revealed marked lipid accumulation, hepatocyte ballooning, and inflammatory cell infiltration. However, CGE treatment, particularly at the high dose, significantly restored body weight, reduced liver index, and alleviated pathological damage, demonstrating its protective effects on liver morphology [30]. Additionally, CGE treatment significantly lowered serum levels of liver injury markers, including AST, ALT, and ALP, indicating its effectiveness in mitigating hepatocyte damage [31]. CGE also suppressed alcohol-induced inflammatory responses by significantly downregulating key inflammatory mediators, such as TNF-α, IL-1β, and CXCL-10, thereby further reducing liver injury [32]. More importantly, CGE exhibited strong antioxidant effects by enhancing the body’s antioxidant defense system, thereby alleviating oxidative stress. Specifically, CGE significantly increased T-AOC and T-SOD levels while reducing lipid peroxidation products (MDA). Lipid peroxidation is a chain reaction driven by free radicals, leading to the formation of lipid hydroperoxides as primary products, which can further decompose into secondary products such as MDA. Elevated concentrations of MDA can exacerbate oxidative stress by forming adducts with proteins and DNA, disrupting their normal function, impairing cellular processes, and contributing to the development of various pathological conditions such as inflammation and tissue damage [33]. These findings suggest that CGE mitigates lipid peroxidation by boosting antioxidant enzyme activity and reducing free radical levels, ultimately suppressing alcohol-induced oxidative stress [34]. Moreover, the results indicated a dose-dependent pharmacological effect of CGE, with the high-dose group showing superior efficacy over the low-dose group in restoring body weight, improving liver pathology, reducing inflammation, and enhancing antioxidant capacity. Overall, the findings demonstrate that CGE has the potential to mitigate liver damage and oxidative stress triggered by alcohol consumption.

To uncover the biological mechanisms driving the safeguarding effects of CGE against ALD-related oxidative stress, this study first employed transcriptomics to identify potential targets and signaling pathways. Based on the analysis of transcriptomic sequencing data, the intersection of dDEGs from Mod vs. Con, DEGs from CGE-H vs. Mod, and oxidative stress-related genes revealed 130 oxidative stress-associated differentially expressed genes. The analysis of these genes in the KEGG database identified significant involvement in various pathways, including the MAPK and FOXO signaling cascades. Among the 20 core genes identified, those most closely associated with the Nrf2 antioxidant pathway included *HMOX1*, *CDKN1A*, *RSAD2*, and *THBD*. The regulation of these genes may provide key insights into the antioxidative mechanisms of CGE.

We also employed network pharmacology analysis to further explore the multi-target mechanisms of CGE. Based on the 81 components of CGE identified by UPLC-Q-Orbitrap-MS, a total of 791 potential targets of CGE against ALD-induced oxidative stress were predicted. KEGG pathway analysis of these targets suggested that pathways such as PI3K-Akt, HIF-1, MAPK, cAMP, and FOXO may play critical roles in regulating oxidative stress. By integrating the results of transcriptomic and network pharmacology analyses, we identified the FOXO signaling pathway as a key mechanism through which CGE mitigates ALD-induced oxidative stress. Several key target genes closely associated with this pathway, including *CDKN1A*, *SLC2A4*, *BCL6*, and *TGF-β3*, were selected for further investigation.

*HMOX1* (HO-1) and *CDKN1A* (P21) are key targets of the Nrf2 signaling pathway. As a central antioxidative transcription factor, the role of Nrf2 in mitigating oxidative stress in ALD has been extensively studied [8]. The excessive accumulation of ROS resulting from alcohol metabolism activates Nrf2, causing its dissociation from the inhibitory protein Keap1 and subsequent translocation into the nucleus, where it induces the transcription of antioxidative genes such as HO-1 [35]. HO-1 is a critical hepatoprotective marker that catalyzes heme degradation, producing antioxidative molecules such as biliverdin and carbon monoxide, thereby reducing ROS levels and enhancing cellular antioxidative capacity [36]. In this study, CGE significantly upregulated the gene expression of *HMOX1*. Further experiments demonstrated that CGE markedly increased protein levels of Nrf2 and HO-1 while suppressing Keap1 protein expression. These findings indicate that CGE alleviates oxidative stress associated with ALD by activating the Nrf2/HO-1 signaling pathway. Additionally, *CDKN1A* (P21) plays a synergistic role with Nrf2 in regulating oxidative stress. On the one hand, P21 can competitively bind Keap1 with Nrf2, preventing Nrf2 from being ubiquitinated and degraded, thus activating the Nrf2 pathway and enhancing the antioxidative response [37,38]. On the other hand, under conditions of severe stress, the upregulation of P21 can promote cell cycle arrest and apoptosis, thereby eliminating damaged cells [39]. In this study, CGE downregulated the gene expression of P21 while upregulating its protein expression. This discrepancy may be attributed to post-transcriptional regulation or a negative feedback mechanism between transcription and translation [40], which warrants further investigation to elucidate its precise biological significance. These results suggest that CGE exerts antioxidative effects by activating the P21/Nrf2/HO-1 signaling axis, thereby ameliorating ALD.

In addition to the P21/Nrf2/HO-1 axis, CGE mitigates alcohol-induced oxidative stress by regulating multiple genes, including RSAD2, BCL6, THBD, SLC2A4, and TGF-β3. *RSAD2* helps maintain cellular redox balance by inhibiting mitochondrial fatty acid oxidation and ROS production [41]. Similarly, BCL6 reduces ROS levels by suppressing Nicotinamide Adenine Dinucleotide Phosphate (NADPH) oxidase expression, thereby alleviating oxidative stress [42,43]. Additionally, the downregulation of SLC2A4 may reduce glucose uptake and metabolism, leading to decreased ROS production [44,45]. THBD exhibits dual regulatory functions. Some studies suggest that it alleviates oxidative stress by increasing antioxidant enzyme activity [46,47], while other studies indicate that antagonizing THBD can alleviate liver fibrosis by clearing senescent cells [48,49]. Therefore, the downregulation of THBD in the context of ALD-induced oxidative stress requires further investigation. Furthermore, the downregulation of TGF-β3 may reduce ROS generation by inhibiting NADPH oxidase activity [50]. These findings highlight the multi-target regulatory effects of CGE in ALD, warranting further investigation.

In summary, this study employed a combined transcriptomics and network pharmacology approach to demonstrate that CGE alleviates ALD-associated oxidative stress by activating the P21/Nrf2/HO-1 signaling axis and modulating a multi-gene network. This multi-target mechanism provides a scientific foundation for the potential use of CGE in the treatment of ALD. In previous studies, our group isolated sesquiterpenoid active compounds, lactucin, and lactucopicrin, from CGE. Additionally, the present study identified a broad range of terpenoid compounds in CGE, suggesting that future research could focus on investigating the bioactivity of these terpenoids. Furthermore, integrating metabolomic and proteomic approaches could help validate the multidimensional mechanisms of CGE and explore its potential applications in other oxidative stress-related diseases. Such investigations will contribute to a more comprehensive understanding of the medicinal value of CGE and its primary components. This research framework also offers valuable insights for the development of natural products and novel strategies for ALD treatment.

## 5. Conclusions

This study reveals that CGE mitigates oxidative stress in ALD mice through the activation of the P21/Nrf2/HO-1 pathway and the modulation of gene expression for *RSAD2*, *BCL6*, *THBD*, *SLC2A4*, and *TGFβ3*, thereby establishing a multi-target synergistic protective mechanism against ALD. Additionally, we identified the components of CGE for the first time, revealing that terpenoids are the most abundant compounds in CGE. This suggests that the anti-oxidative stress effect of CGE against ALD-induced oxidative stress is likely primarily attributable to the presence of terpenoids. Our results offer a robust groundwork for utilizing *Cichorium glandulosum* as a natural antioxidant and highlight its potential applications in ALD and other disorders associated with oxidative stress.

## Data Availability

The original contributions presented in the study are included in the article and Supplementary Materials. Further inquiries can be directed to the corresponding authors.

## Supplementary Materials

The following supporting information can be downloaded at: https://www.mdpi.com/article/10.3390/metabo15010041/s1. Table S1: Gradient Elution Conditions; Table S2: Mass Spectrometry Parameters; Table S3: Identification of Chemical Constituents in the Ethyl Acetate extract of Cichorium glandulosum; Table S4: Primers for RT-qPCR.
