# Supplementary material for: Ethyl Acetate Extract of Cichorium glandulosum Activates the P21/Nrf2/HO-1 Pathway to Alleviate Oxidative Stress in a Mouse Model of Alcoholic Liver Disease
Source: Metabolites. 2025 Jan 10;15(1):41. doi: 10.3390/metabo15010041 (PMC11767034; doi:10.3390/metabo15010041)
Supplement: Supplementary file 1 [file metabolites-15-00041-s001.zip › Supplementary Materials.docx]

**Table S1** Gradient Elution Conditions

| **Time(min)** | **A%** | **B%** |
| --- | --- | --- |
| 0 | 95 | 5 |
| 2 | 95 | 5 |
| 4 | 70 | 30 |
| 8 | 50 | 50 |
| 10 | 20 | 80 |
| 14 | 0 | 100 |
| 15 | 0 | 100 |
| 15.1 | 95 | 5 |
| 16 | 95 | 5 |

**Table S2** Mass Spectrometry Parameters

| **Parameter** | **Positive Ion** | **Negative Ion** |
| --- | --- | --- |
| Spray Voltage (V) | 3800 | -3000 |
| Capillary Temperature (°C) | 320 | 320 |
| Aux gas heater temperature (℃) | 350 | 350 |
| Sheath Gas Flow Rate (Arb) | 35 | 35 |
| Aux gas flow rate (Arb) | 8 | 8 |
| S-lens RF level | 50 | 50 |
| Mass range (m/z) | 100-1500 | 100-1500 |
| Full ms resolution | 60000 | 60000 |
| MS/MS resolution | 15000 | 15000 |
| NCE/stepped NCE | 10，20，40 | 10，20，40 |
| Aux gas heater temperature (℃) | 350 | 350 |
| Sheath Gas Flow Rate (Arb) | 35 | 35 |
| Aux gas flow rate (Arb) | 8 | 8 |

**Table S3** Identification of Chemical Constituents in the Ethyl Acetate Extract of *Cichorium glandulosum*

| NO. | t_R_(min) | Identify Components | Formula | Adducts | Fragment Ion | Chemical Class |
| --- | --- | --- | --- | --- | --- | --- |
| 1 | 1.835 | Gallic acid | C7H6O5 | M-H | 125.0241, 169.0138 | Phenols |
| 2 | 3.303 | 3,5-Dihydroxybenzoic acid | C7H6O4 | M-H | 109.0302, 153.0188 | Carboxylic acid and Derivatives |
| 3 | 4.140 | 4-hydroxy-2 hexenoic acid | C6H10O3 | M+FA-H | 85.0657, 113.0606, 115.0398, 131.071, 146.9609, 157.0502, 175.0608 | Fatty Acyls |
| 4 | 4.201 | Gentisic acid | C7H6O4 | M-H | 108.0215, 109.0293, 151.0033, 153.0189 | Organic acids and derivatives |
| 5 | 4.301 | Dihydrocaffeic acid | C9H10O4 | M-H | 59.0137, 109.0292, 109.0657, 119.0499, 121.0293, 135.0447, 137.0605, 166.0266, 181.0501 | Phenylpropanoids |
| 6 | 4.301 | Methyl 5-hydroxypyridine-2-carboxylate | C7H7NO3 | M-H | 152.0349 | Alkaloids |
| 7 | 4.339 | Phthalic acid | C8H6O4 | M-H | 93.0344, 121.0292, 135.0449, 139.0401, 165.0189 | Organic acids and derivatives |
| 8 | 4.437 | Esculetin | C9H6O4 | M-H | 149.0240, 163.2953, 166.8896, 171.8446, 175.9422, 175.9823, 176.4226, 176.7810, 176.8810, 177.0188 | Phenylpropanoids |
| 9 | 4.674 | 3-Feruloylquinic acid | C17H20O9 | M-H | 93.0344, 173.0451, 191.0556, 193.05, 367.102 | Phenylpropanoids |
| 10 | 4.712 | 3-O-p-coumaroylquinic acid | C16H18O8 | M-H | 93.0344, 163.0396, 173.0451, 191.0557, 337.0536, 337.0923 | Carboxylic Acids |
| 11 | 4.750 | Feroxidin | C11H14O3 | M+FA-H | 151.1124, 154.0252, 177.0915, 179.0702, 195.0117, 195.0672, 195.1020, 195.1368, 239.0563, 239.0917 | Phenols |
| 12 | 4.806 | (2*R*)-2-butoxybutanedioic acid | C8H14O5 | M-H | 99.0813, 127.0762, 129.0554, 145.0867, 171.0661, 189.0763 | Organic acids and derivatives |
| 13 | 4.902 | Suberic acid | C8H14O4 | M-H | 111.0813, 129.0918, 173.0814 | Fatty Acyls |
| 14 | 4.921 | Isoquercitrin | C21H20O12 | M-H | 271.024, 300.0268, 463.0867 | Flavonoids |
| 15 | 4.921 | (*E*)-P-Coumaricacid | C9H8O3 | M-H | 119.0499, 163.0397 | Phenylpropanoids |
| 16 | 4.960 | Ethyl gallate | C9H10O5 | M-H | 125.0241, 169.0138, 197.045 | Phenols |
| 17 | 4.960 | Ellagic acid | C14H6O8 | M-H | 300.9984 | Phenols |
| 18 | 5.016 | 3-(3-Hydroxyphenyl)propanoic acid | C9H10O3 | M-H | 72.993, 93.0345, 119.05, 121.0293, 121.0656, 147.0448, 151.0033, 165.0216, 165.0552 | Organic acids and derivatives |
| 19 | 5.055 | 1-Deoxyeucommiol | C9H16O3 | M+FA-H | 155.1074, 171.1027, 217.1075 | Terpenes |
| 20 | 5.111 | Isoferulic acid | C10H10O4 | M-H | 134.037, 137.0242, 149.0604, 149.0966, 178.0266, 192.9958, 193.0137, 193.05 | Phenylpropanoids |
| 21 | 5.129 | Quercitrin | C21H20O11 | M-H | 151.0398, 175.0395, 255.0293, 284.032, 285.0396, 447.0907 | Flavonoids |
| 22 | 5.129 | Kaempferol 3-O-β-D-glucuronide | C21H18O12 | M-H | 113.0242, 175.0243, 285.0397, 461.071 | Flavonoids |
| 23 | 5.168 | Isorhamnetin-3-O-β-glucoside | C22H22O12 | M-H | 243.0291, 271.024, 285.0397, 314.0424, 477.1029 | Flavonoids |
| 24 | 5.263 | 11,13-dihydrolactucin | C15H18O5 | 2M-H | 555.2235,259.0292,  215.1025 | Terpenes |
| 25 | 5.397 | Trilobatin | C21H24O10 | M-H2O-H | 297.0759, 417.1194 | Flavonoids |
| 26 | 5.434 | 6-Oxooctanoic acid | C8H14O3 | M+FA-H | 59.0137, 72.993, 113.097, 141.0918, 143.0708, 185.0812, 203.0919 | Organic acids and derivatives |
| 27 | 5.568 | L-α-Hydroxyarbusculin A | C15H22O4 | M-H | 87.0084, 247.1323, 265.0706, 265.1069, 265.1427 | Terpenes |
| 28 | 5.721 | Ethyl 3,4-dihydroxybenzoate | C9H10O4 | M-H | 109.0292, 137.0968, 151.0033, 152.011, 153.019, 166.0267, 181.0503 | Carboxylic acid and derivatives |
| 29 | 5.739 | 8-deoxylactucin | C15H16O4 | M+FA-H | 259.0215, 259.0974, 261.0393, 261.0767, 261.1119, 261.1472, 269.1050, 269.1390, 287.0925, 305.0249 | Terpenes |
| 30 | 5.971 | 3,4,5-Trimethoxyhydrocinnamic acid | C12H16O5 | M-H | 137.0241, 149.0604, 193.0501, 239.092 | Phenylpropanoids |
| 31 | 6.109 | Coronopolin | C15H20O4 | M-H | 125.0605, 139.0762, 151.0761, 152.084, 153.0918, 201.1281, 204.115, 219.1386, 263.0921, 263.1279 | Terpenes |
| 32 | 6.129 | Torachrysone-8-O-β-D-glucoside | C20H24O9 | M-H | 230.0578, 245.0814, 407.1324 | Quinones |
| 33 | 6.210 | Morin | C15H10O7 | M-H | 151.0033, 178.9982, 301.0347 | Flavonoids |
| 34 | 6.433 | Vaccaxanthone | C16H12O8 | M-H2O-H | 269.045, 313.0348 | Phenylpropanoids |
| 35 | 6.495 | Ethyl Caffeic acid | C11H12O4 | M-H | 135.0449, 161.0241, 179.0346, 207.0657 | Phenylpropanoids |
| 36 | 6.535 | 5-(2,5-dihydrofuran-3-yl)-2-methylPent-3-en-2-ol | C10H16O2 | M+FA-H | 151.1124, 169.123, 213.0915, 213.1125 | Terpenes |
| 37 | 6.594 | 11β,13-Dihydrolactucopicrin | C23H24O7 | M-H | 215.1072, 216.1105, 259.0970, 260.1003, 277.1072, 278.1109, 349.1436, 365.1947, 393.1335, 411.1421 | Terpenes |
| 38 | 6.636 | Lactucopicrin | C23H22O7 | M-H | 107.05, 151.0398, 185.0969, 195.0808, 198.0682, 213.0917, 239.0707, 257.0814, 275.092, 409.1278 | Terpenes |
| 39 | 6.837 | Olibanumol B | C10H18O2 | M+FA-H | 87.0086, 153.1281, 197.1179, 215.1283 | Terpenes |
| 40 | 7.018 | Kaempferol | C15H10O6 | M-H | 285.0398 | Flavonoids |
| 41 | 7.223 | Traumatic acid | C12H20O4 | M-H | 165.128, 183.1385, 227.1283 | Fatty Acyls |
| 42 | 7.672 | Isotrifoliol | C16H10O6 | M-H | 253.05, 297.0397 | Flavonoids |
| 43 | 9.979 | Emodin | C15H10O5 | M-H | 269.045 | Quinones |
| 44 | 12.167 | 3-Hydroxypalmitic acid | C16H32O3 | M-H | 225.2222, 271.2271 | Fatty Acids |
| 45 | 4.091 | 3,4-Dihydroxybenzaldehyde | C7H6O5 | M+H | 65.0391, 93.0337, 111.0441, 139.0386 | Phenols |
| 46 | 4.383 | Salicyl alcohol | C_7_H_6_O_3_ | M+H-H2O | 79.0546, 95.0494, 107.0492 | Phenols |
| 47 | 4.474 | Caffeic acid | C_7_H_8_O_2_ | M+H-H2O | 107.0492, 133.0281, 135.0437, 139.5287, 145.028, 163.0381 | Organic acids and derivatives |
| 48 | 4.492 | Paeonilactone B | C_9_H_8_O_4_ | M+H | 73.0288, 125.0595, 151.0386, 151.0745, 179.0697, 197.0805 | Terpenes |
| 49 | 4.700 | 3-Hydroxybenzaldehyde | C_11_H_14_O_5_ | M+H | 67.0546, 81.0703, 95.0494, 95.0857, 123.0442 | Phenols |
| 50 | 4.924 | Quercetin 3-O-β-D-glucofuranoside | C_7_H_6_O_2_ | M+H | 303.0488 | Flavonoids |
| 51 | 4.981 | Lactucin | C21H20O12 | M+Na | 255.0618, 299.0877 | Terpenes |
| 52 | 4.981 | Isovanillin | C_15_H_16_O_5_ | M+H | 65.0391, 93.0337, 107.0856, 110.0364, 111.0441, 125.0595, 135.08, 153.0542 | Phenols |
| 53 | 5.106 | Syringaldehyde | C_11_H_10_O_4_ | M+H | 95.0494, 113.9636, 123.0439, 123.0801, 137.0958, 140.0465, 155.0699, 165.0905, 183.0647 | Phenols |
| 54 | 5.106 | Ferulic acid | C_9_H_10_O_4_ | M+H | 117.0335, 145.028, 149.0593, 163.0385, 177.0541 | Organic acids and derivatives |
| 55 | 5.125 | Isochlorogenic acid C | C_10_H_10_O_4_ | M+H-H2O | 163.0385, 319.08, 499.1218 | Phenylpropanoids |
| 56 | 5.161 | Scopoletin | C_25_H_24_O_12_ | M+H | 133.0282, 193.0491 | Phenylpropanoids |
| 57 | 5.179 | Brassicin | C_10_H_8_O_4_ | M+H | 317.0645 | Flavonoids |
| 58 | 5.252 | Austricine | C_22_H_22_O_12_ | M+H | 199.1114, 203.1058, 217.1215, 227.1053, 227.1268, 245.1162, 245.1369, 263.0526, 263.0888, 263.1262 | Terpenes |
| 59 | 5.342 | Britannilactone | C_10_H_14_O_3_ | M+H | 149.0954, 157.1006, 175.1112, 175.1474, 185.1321, 203.1424, 231.1373, 249.1467, 267.1190, 267.1553 | Terpenes |
| 60 | 5.381 | Aloeresin D | C_15_H_22_O_4_ | M+H | 247.0956, 301.1063, 309.0954, 331.1164, 349.1266, 393.1314, 419.1478, 437.1582, 513.1740, 557.1996 | Quinones |
| 61 | 5.418 | Isololiolide | C_29_H_32_O_11_ | M+H | 107.0856, 133.1009, 135.1165, 161.0955, 179.106, 197.1164 | Terpenes |
| 62 | 5.418 | Ononin | C_11_H_16_O_3_ | M+H | 261.0411, 269.08, 413.1241, 431.1278 | Flavonoids |
| 63 | 5.581 | Sec-hydroxyaegineticacid | C_22_H_22_O_9_ | M+Na | 151.0749, 261.1844, 271.1681, 289.1786, 307.0599, 307.1173, 307.1513 | Norcarotenoids |
| 64 | 5.581 | Sinapaldehyde | C15H24O5 | M+H | 153.0542, 163.0755, 163.1112, 173.0957, 177.0542, 181.0853, 191.0698, 191.1058, 191.1417, 209.0802 | Phenylpropanoids |
| 65 | 5.636 | Barrelin | C_11_H_12_O_4_ | M+H | 173.0957, 173.1318, 187.1111, 191.1063, 201.1268, 205.1217, 219.1375, 229.1217, 247.1319, 265.1412 | Terpenes |
| 66 | 5.804 | 3,4-Dimethoxycinnamic acid | C_15_H_20_O_4_ | M+H-H2O | 163.1113, 191.0697 | Phenylpropanoids |
| 67 | 5.901 | Isorhapontigenin | C_11_H_12_O_4_ | M+H | 167.0849, 169.1006, 183.0798, 185.0954, 189.0533, 195.08, 213.0903, 217.0492, 241.0851, 259.0957 | Phenylpropanoids |
| 68 | 6.096 | Chinensiolide B | C_15_H_14_O_4_ | M+H-H2O | 201.0905, 201.1268, 205.0848, 205.1213, 211.1109, 219.1375, 229.0859, 229.1216, 247.0941, 247.1318 | Terpenes |
| 69 | 6.196 | Quercetin | C_15_H_20_O_4_ | M+H | 303.049 | Flavonoids |
| 70 | 6.236 | Calycosin | C_15_H_10_O_7_ | M+H | 270.0524, 253.0570,  225.0545 | Flavonoids |
| 71 | 6.295 | Syringaresinol | C_16_H_12_O_5_ | M+H-H2O | 315.0851, 323.1262, 330.1084, 339.1219, 343.1160, 351.1214, 369.1324, 371.1481, 383.1480, 401.1595 | Phenylpropanoids |
| 72 | 6.614 | Pseudolaric Acid C | C_15_H_22_O_4_ | M+Na | 197.0956, 198.0991, 215.1061, 216.1094, 233.1160, 243.1009, 244.1045, 261.1112, 262.1145, 413.1158 | Terpenes |
| 73 | 6.635 | 2,3,11,13-Tetrahydrohelenalin | C_21_H_26_O_7_ | M+Na | 289.1393 | Terpenes |
| 74 | 6.794 | 4α,5α-epoxy-6α-hydroxyamorphan-12-oic acid | C15H22O4 | M+Na | 273.1837, 291.1197, 291.156 | Terpenes |
| 75 | 7.295 | Santonin | C_15_H_24_O_4_ | M+H | 145.1007, 173.0956, 174.1031, 183.1164, 201.1268, 229.1216, 247.1319 | Terpenes |
| 76 | 7.576 | 11β,13-dihydroreynosin | C_15_H_18_O_3_ | M+H-H2O | 119.0855, 131.0853, 145.1008, 157.1008, 159.1164, 177.0905, 187.1476, 215.1423, 233.1154, 233.1527 | Terpenes |
| 77 | 8.216 | Blumenol C | C15H22O3 | M+NH4 | 137.0959, 147.1167, 149.1321, 151.1479, 175.1479, 193.1585, 211.1679, 228.1013, 228.1586, 228.1964 | Terpenes |
| 78 | 9.339 | Dehydrophytosphingosine | C_13_H_22_O_2_ | M+H | 60.0449, 280.262, 298.273, 316.2837 | Sphingolipids |
| 79 | 9.482 | Piperine | C_18_H_37_NO_3_ | M+H | 135.0437, 201.0541, 286.1427 | Alkaloids |
| 80 | 9.562 | Nardosinone | C_17_H_19_NO_3_ | M+NH4 | 95.0859, 159.1157, 177.1634, 187.1477, 189.1632, 205.1583, 233.1527, 251.1633, 268.1898 | Terpenes |
| 81 | 9.624 | Phytosphingosine | C_15_H_22_O_3_ | M+H | 60.045, 282.2782, 300.289, 318.2993 | Sphingolipids |

**Table S4** Primers for RT-qPCR

| Gene | Species | Forward Primer | Reverse Primer |
| --- | --- | --- | --- |
| HMOX1 | Mouse | TCCTTGTACCATATCTACACGG | GAGACGCTTTACATAGTGCTGT |
| CDKN1A | Mouse | ATGTCCAATCCTGGTGATGTC | GAAGTCAAAGTTCCACCGTTC |
| THBD | Mouse | GCTGTGAGTACTTGTGCAATAG | TCACACATACAGGAGTAAGAGC |
| RSAD2 | Mouse | AGTGTCAACTACCACTTCACTC | GGTTCTCCTCCAGAAAAGTTGA |
| SLC2A4 | Mouse | TATTCAACCAGCATCTTCGAGT | GTCCAGCTCGTTCTACTAAGAG |
| BCL6 | Mouse | AACCATACAAATGTGATCGCTG | CACAAATGTTACAGCGATAGGG |
| TGFβ3 | Mouse | ATCAAGAAGAAGAGGGTGGAAG | GTAAAGTGCCAGGACCTGATAG |
